# Cooking up Change: DEIB Principles as Key Ingredients in Nutrition and Culinary Medicine Education

**DOI:** 10.3390/nu15194257

**Published:** 2023-10-05

**Authors:** Melinda Ring, David Ai, Geeta Maker-Clark, Raeanne Sarazen

**Affiliations:** 1 Osher Center for Integrative Health, Feinberg School of Medicine, Northwestern University, Chicago, IL 60611, USA; 2 Baylor College of Medicine, Houston, TX 77030, USA; david.ai@live.com; 3 NorthShore University Health System, Pritzker School of Medicine, University of Chicago, Chicago, IL 60637, USA; geetamaker@gmail.com; 4 Academy of Nutrition and Dietetics, Chicago, IL 60606, USA; raeanne@raeannesarazen.com

**Keywords:** diversity, equity, inclusion, belonging, nutrition education, cultural competence, culinary medicine, medical education, culturally sensitive care

## Abstract

The integration of diversity, equity, inclusion, and belonging (DEIB) principles into healthcare education is essential to ensure culturally sensitive and equitable healthcare delivery. In the domain of nutrition, food, and health, these principles are particularly vital, as diet and food choices are strongly linked to cultural identities and socioeconomic conditions. Despite a growth of DEIB initiatives in undergraduate and graduate medical education, there is a significant gap regarding guidelines for implementing DEIB principles in education around nutrition and food, including that for dietitians, allied health and medical professionals. A literature review was conducted, analyzing peer-reviewed articles and current practices in academic medical education to understand DEIB in nutrition, food, and health. The outcome was the creation of a three-tiered checklist titled “Checklist for Culturally Competent Education in Nutrition”. It serves as a roadmap to cultivate culturally competent, equitable, and inclusive healthcare professionals that emphasizes avoiding bias, enhancing awareness, and building practical skills for DEIB implementation around nutrition.

## 1. Introduction

The evidence for the role of what we eat in health outcomes is undeniable and ever-growing. As our society becomes increasingly diverse, it becomes all the more critical for healthcare professionals to have a strong understanding of diversity, equity, inclusion, and belonging (DEIB) principles. In the realm of nutrition, food, and health, these principles are particularly vital, as diet and food choices are intimately intertwined with cultural identities, socioeconomic conditions, and personal beliefs.

In recent years, there has been a notable surge in medical education initiatives aimed at incorporating diversity, equity, inclusion, and belonging (DEIB) principles into undergraduate and graduate medical education [1,2,3,4,5,6,7,8,9,10]. Concurrently, the development of culinary medicine curricula, which synergistically blend nutrition, food science, and health, has been on the rise [11,12,13,14,15,16]. A commendable resource that has been embraced by numerous institutions is “The Upstate Bias Checklist: A Checklist for Assessing Bias in Health Professions Education Content” [17]. However, when considering its application in the authors’ health professional courses focusing on nutrition, several significant gaps were identified. This prompted a deeper exploration into the creation of a checklist that more adequately addresses the intricacies of cultural competency in the realm of food and nutrition.

In our pursuit to identify a tool that encapsulates the critical DEIB principles pertaining to food, eating, and health, we embarked on a comprehensive literature review. This endeavor highlighted a pronounced gap in the existing literature; as of now, there are no established guidelines available for faculty and chef-instructors to facilitate the implementation of DEIB principles in nutrition education. Addressing this void is paramount to ensuring that our healthcare professionals are adept at providing culturally sensitive and equitable healthcare. The ultimate objective is to foster positive lifestyle changes that significantly enhance the health and well-being of both individuals and communities. Therefore, it is imperative that DEIB principles are woven into the very fabric of our educational curricula on nutrition.

Our analysis of diversity, equity, inclusion, and belonging (DEIB) within the domains of nutrition, food, and health has illuminated the multifaceted and extensive nature of this crucial area of study. Bearing these insights in mind, we have formulated a proposed checklist. This tool is envisioned to steer the seamless integration of DEIB principles into the pertinent curricula, thereby nurturing a generation of health professionals who are well-versed in these essential values.

## 2. Methods

To comprehensively explore the topic of diversity, equity, inclusion, and belonging (DEIB) in the spheres of nutrition, food, and health, we initiated a narrative literature review. This search was conducted using the PubMed and Google Scholar databases, with a selection of specific keywords devised to encompass the full scope and complexity of the subject matter. These terms, including “diversity”, “equity”, “inclusion”, “belonging”, “nutrition education”, “culinary medicine”, and “cultural competence”, among others, were employed in various combinations to pinpoint pertinent articles.

Parallel to the literature review, we scrutinized the prevailing academic medical education strategies for incorporating DEIB elements into the curriculum. This process entailed a detailed analysis of the curricular frameworks, pedagogical methodologies, and evaluation strategies utilized by a subset of medical schools and healthcare education institutions to integrate DEIB principles into their respective programs. These institutions were selected for their contributions to academic literature on DEIB in recent years, their affiliation with the authors, and their participation in a multi-center initiative led by the Osher Collaborative for Integrative Health to incorporate DEIB principles into existing curricula.

Following the identification of the most germane references, we assembled a collection of sixty-five peer-reviewed research articles, reviews, policy documents, commentaries, and reports that concentrate on DEIB within the realms of nutrition, culinary medicine, and health education. This compilation was subjected to a rigorous analysis by two independent reviewers (M.R., D.A.), who identified common themes and insights, subsequently categorizing the data into distinct sectors such as Access to Food, Body Image, Cultural Humility, Gender and Sexuality, Language and Stereotyping, Self-Efficacy, Stigma associated with Food and Diet, Weight Bias, and DEIB initiatives in Health Professional, Nutrition, and Dietary Education. To synthesize the varied literature into a unified understanding of the topic, we adopted a narrative synthesis approach.

This dual approach of literature review and examination of contemporary practices in medical education furnished a comprehensive panorama of the current landscape of DEIB in nutrition, food, and health education. This robust groundwork facilitated the subsequent creation of a competency checklist, catering to the unique and multifaceted needs of this critical area in healthcare education. While we recognize the value of providing a detailed methodology and listing the specific documents reviewed, we wish to clarify that the primary aim of this article is to introduce a DEIB-focused checklist for educators. A more exhaustive scoping review is planned for a separate, future article.

## 3. Results

Upon completing the literature review and analysis, the critical areas pertinent to the integration of diversity, equity, inclusion, and belonging (DEIB) within the context of education in nutrition were systematically organized into three overarching categories to serve as a roadmap to cultivate learning environments where future healthcare professionals can become proficient in delivering culturally competent, equitable, and inclusive care. These categories are aimed at guiding a comprehensive understanding and practical application of DEIB principles:

I. DEIB in Nutrition Curriculum Content (Table 1)
  A. Health Equity
  B. Language
  C. Cultural Humility
  D. Representation
II. DEIB in Recipes and Cooking (Table 2)
  A. Cultural Representation and Respect
  B. Accessibility
  C. Dietary Inclusion
  D. Language Sensitivity
III. DEIB in Curriculum Delivery (Table 3)
  A. Safe Space for Dialogue
  B. Goal Setting and Evaluation
  C. Continuous Improvement
  D. Instructional Methods and Accessibility

Furthermore, the “Checklist for Culturally Competent Education in Nutrition” provides actionable steps across three progressively more engaged tiers:

Tier 1: Avoiding Bias and Discrimination—This foundational step focuses on the identification and elimination of any existing biases or discriminatory practices within the curriculum. The objective here is to create a safe and inclusive learning environment, where no harm comes from prejudice or stereotyping.

Tier 2: Enhancing DEIB and Cultural Awareness—Building upon the first tier, this phase emphasizes the integration of diversity, equity, inclusion, and belonging principles into the teaching and learning experience. The goal is to increase awareness and understanding of different cultures, identities, and perspectives, fostering empathy and respect among students.

Tier 3: Building Skills for DEIB Implementation—The final tier is geared towards actively developing the practical skills needed to implement DEIB principles in a professional healthcare setting. It not only encourages critical thinking about DEIB but also supports learners in translating these principles into their future practice. It ensures that future healthcare professionals are equipped not only with theoretical understanding but also with the tools to apply these principles in real-world scenarios.

Each tier on this checklist represents a vital component of our overarching objective to deliver health care that respects and acknowledges the diverse needs, experiences, and perspectives of all patients. The sequence of these tiers represents a journey from avoiding harm to actively promoting and implementing diversity and inclusivity. It reflects a commitment to more than just compliance with rules, moving towards a genuine embrace of cultural diversity and equitable practices. This checklist is not just a tool, but a commitment to nurturing a new generation of health professionals who not only understand the essential role of nutrition in health but are also deeply committed to applying DEIB principles in their practice.

## 4. Discussion

Globally, there is an escalating urgency to address issues of nutrition, health, and equity across all sectors. Noteworthy developments in this regard in the U.S. include the following:

- Recent initiatives spearheaded by the U.S. government, such as the White House Challenge to End Hunger and Build Healthy Communities [18] and a bipartisan house resolution urging medical schools, residency, and fellowship programs to elucidate the link between diet and disease through education on nutrition [19];
- The American Medical Association’s recent policy shift, which acknowledges the historical harm associated with using the body mass index (BMI) as a measurement tool [20];
- A collaborative effort by major medical education organizations to host a Summit on Medical Education in Nutrition; convened by ACGME (Accreditation Council for Graduate Medical Education), AACOM (American Association of Colleges of Osteopathic Medicine), and AAMC (Association of American Medical Colleges) [21].

The inception and development of the “Checklist for Culturally Competent Education in Nutrition” accentuate the pressing need to cultivate DEIB principles within healthcare, specifically in the area of education around nutrition and culinary medicine. While higher education programs are gradually incorporating related concepts, and similar methodologies have been employed in university courses focusing on sustainable food systems, a broader application in the training of those professionals who will be caring for patients remains to be seen [22,23].

A nuanced understanding of dietary needs and health outcomes necessitates the consideration of cultural identities, social determinants, and individual preferences. Our initiative has spotlighted the intricate relationship between these elements, urging healthcare professionals to adopt a holistic approach to nutrition and health. This strategy fosters cultural competency and inclusivity in healthcare education, addressing specific medical and dietary conditions such as food allergies, intolerances, and orthorexia, while also rectifying historically culturally insensitive frameworks, like the universal prescription of the Mediterranean Diet. 

The proposed checklist transcends theoretical knowledge, offering actionable steps and practical skills that equip healthcare professionals to integrate DEIB principles into their practice. Grounded in contemporary research and best practices in the realms of DEIB and nutrition, the checklist is poised to remain relevant amidst the swiftly evolving landscape of healthcare.

Moreover, the checklist is customized to meet the distinctive challenges of nutrition, food, and health education, serving as a precious resource for both existing and emerging curricula. It offers guidance to faculty members, chef-instructors, and curriculum developers, with the versatility to adapt to various educational environments, including traditional classrooms, online platforms, and experiential learning or teaching kitchens. This adaptability facilitates the seamless incorporation of DEIB principles into curricula, irrespective of the specific context or setting.

Furthermore, the checklist can be utilized flexibly, either as a structured step-by-step guide or as a broad framework to inform educators ranging from curriculum developers to teachers. By adopting specific strategies and resources to uplift underrepresented and marginalized groups in healthcare education, it aims to foster equitable access and opportunities, thereby addressing implicit bias in this domain.

While the checklist lays a solid foundation, it necessitates further empirical testing and validation across diverse educational landscapes. Collaborative efforts with various stakeholders, coupled with ongoing refinement through a planned modified Delphi process, will ensure its sustained relevance and efficacy.

## 5. Conclusions

The integration of DEIB principles into nutrition and culinary education transcends a moral imperative; it delineates a route towards a more compassionate, effective, and culturally attuned healthcare system. Through our extensive review, we unearthed critical insights from a rich tapestry of literature and existing curricular approaches, which have significantly informed the creation of our checklist. Our research underscored the pervasive issues of access to food, body image perceptions, and the necessity for cultural humility in healthcare settings, among other themes.

Drawing from a diverse array of sources, we have recognized the pressing need to address gender and sexuality biases, language and stereotyping, and stigma associated with food and diet in educational initiatives. These findings have been instrumental in shaping a tool that is not only grounded in current research but also reflective of the complex interplay of factors that influence health outcomes.

This review and the resultant checklist stand as a seminal resource, poised to mold a future generation of healthcare professionals who embody not only nutritional expertise but also a deep-seated commitment to cultural competence, inclusivity, respect, and equity in patient care. In a world that is increasingly characterized by diverse populations, the call for healthcare that is both culturally sensitive and equitable, and that actively seeks to redress health disparities, is louder than ever.

Our work marks a significant stride towards achieving this vision, presenting a practical and insightful blueprint for the ongoing evolution of professional education in the realms of nutrition, food, and health. It amplifies the call for a comprehensive, inclusive, and adaptable approach to healthcare nutrition education, illustrating that DEIB is not a fleeting initiative but a sustained pledge to fostering equitable and effective healthcare practices.

As we move forward, we remain committed to refining this tool through collaborative efforts and empirical testing, ensuring its adaptability and relevance in a rapidly changing healthcare landscape. This initiative embodies an ongoing dedication to nurturing a healthcare environment that is not only knowledgeable but also empathetic and responsive to the diverse needs of communities worldwide.

## Figures and Tables

**Table 1:** DEIB in Nutrition Curriculum Content.

**Health Equity** Health equity, the state in which everyone has a fair and just opportunity to attain their highest level of health, is of paramount importance in educating health professionals on the role of nutrition and food in health. It ensures that future healthcare providers understand and address the diverse dietary needs and health challenges faced by different populations, particularly those affected by social determinants of health.	
Tier 1	Resources and references are up-to-date, reliable, and representative of diverse voices in the field of nutrition. Does not imply biological differences between racial or ethnic groups. Ensures that the educational content remains objective and inclusive, reflecting a diverse array of dietary practices and cultural norms rather than individual biases.
Tier 2	Teaches health professionals to consider social determinants that influence patients’ food choices and access. Addresses disparities in nutritional health within different patient communities. Includes information about legal rights and protection related to food access, discrimination, and health, especially for marginalized patient groups. Embraces and respects the rich diversity of food cultures and traditions pertinent to the patient communities served.
Tier 3	Equips health professionals with strategies to overcome barriers specific to underrepresented or marginalized patient communities. Provides skills and resources to address food access and food security issues among patients. Guides health professionals to additional resources for diverse perspectives and experiences in healthcare. Empowers learners with specialized knowledge to address the unique dietary needs and health challenges of specific populations, including ethnic minorities, immigrants, and low-income communities.
**Language** Language in DEIB is critical as it directly influences how we acknowledge, respect, and validate individual identities and experiences, ultimately fostering an inclusive and equitable environment. It shapes our interactions and has the power to either challenge or perpetuate stereotypes and biases. Using inclusive and respectful language ensures that all individuals and groups are accurately represented and acknowledged, regardless of their background, identity, or culture.	
Tier 1	Employs person-first language, where the individual’s identity precedes the disability (e.g., ‘person with a disability’ instead of ‘disabled person’) to promote respect and avoid stigmatization. Uses inclusive language, such as “they” or “individuals,” to avoid making assumptions about gender. Appreciates that gender identities and pronouns of all persons depicted are varied. Avoids terms that may perpetuate stereotypes related to age, opting instead for neutral expressions like ‘older adults’ or ‘individuals of all ages.’ Eschews the use of stigmatizing or judgmental terminology concerning body size or weight, preferring neutral language that discusses health and wellness without making presumptive judgments about an individual’s health based on appearance. Utilizes language that is accessible to health professionals from various backgrounds. Refrains from labeling individuals with special food needs as “difficult” or “problematic,” and avoids assumptions about an individual’s dietary adherence based on their special food needs. Avoids stigmatizing language such as referring to gluten-free or plant-based diets as “trendy” or “fads,” which can undermine the legitimacy of these needs for some individuals.
Tier 2	Examines the language used for various international dishes and cuisines, fostering an understanding of the cultural significance and richness of diverse food traditions. Promotes intentional discussions around food that avoid sweeping generalizations and stereotypes, acknowledging the historical and cultural nuances of specific dishes and culinary practices, and promoting a more comprehensive and respectful understanding of food cultures. Emphasizes the importance of patient preference in word choices that may be perceived as stigmatizing, such as “fat” or “obese,” advocating for respectful communication.
Tier 3	Teach health professionals to use patient-centered language in nutrition counseling. Trains health professionals to promote a positive relationship with food and body image among patients.
**Cultural Humility** Cultural humility, a process of self-reflection and self-critique, is an essential component of an equitable and inclusive nutrition curriculum. It guides health professionals to acknowledge and respect the diverse food cultures and dietary practices of their patients. By fostering cultural humility, professionals can better understand the complexities of nutrition within different cultural contexts, and avoid a one-size-fits-all approach, thereby promoting personalized, effective, and respectful patient care.	
Tier 1	Resources and references are up-to-date, reliable, and representative of diverse voices in the field. Does not imply biological differences between racial or ethnic groups.
Tier 2	Equips professionals to understand different cultural perspectives on food and nutrition. Includes diverse dietary guidelines and nutrition advice relevant to all communities served. Respects and includes indigenous knowledge and traditional food practices relevant to healthcare. Incorporates input from health professionals from various cultural backgrounds in content development.
Tier 3	Encourages health professionals to recognize and reflect on their own identities and implicit biases, understanding how these factors may impact patient counseling around weight and nutrition. Allows health professionals to share their experiences of cultural diversity in healthcare settings.
**Representation** Representation refers to ensuring that individuals from a wide range of backgrounds, identities, and experiences are involved in the design, implementation, and evaluation of the DEIB-focused nutrition curriculum. By having such representation, the curriculum can be informed by a variety of perspectives, enhancing its cultural relevance, inclusivity, and effectiveness. Moreover, it promotes an environment where all voices are valued and can contribute to shaping the educational experience.	
Tier 1	Diverse voices and perspectives are represented in course materials, including patient stories and images. Includes diverse instructors in terms of backgrounds and areas of expertise.
Tier 2	Involves community organizations and stakeholders in the development of the curriculum. Discusses the role of cultural competence, representation, and empathy in providing effective and respectful nutrition guidance. Includes comprehensive information on the range of special food needs such as food allergies, intolerances, celiac disease, religious dietary restrictions, and medical diet restrictions. Teaches health professionals about the grief, loss, and identity change that may accompany a new diagnosis of a diet-related condition (e.g., celiac disease), emphasizing the importance of empathy and support. Educates health professionals on how stigma negatively impacts individuals with special food needs both psychologically and socially, such as isolation, diminished bonding over food, lack of spontaneity, and cost considerations, fostering a more compassionate approach. Incorporates content about the different nutritional needs across the gender spectrum, considering factors such as hormonal variations, reproductive health, and risk for certain diseases. Recognizes and discusses the societal pressures and expectations around body image and eating behaviors for different genders, and their impact on nutrition and health. Refrains from stereotyping dietary behaviors based on gender or sex, such as assuming men eat more protein or women are more likely to diet.
Tier 3	Incorporates experiential learning opportunities that expose students to diverse populations and their unique nutritional needs and challenges, such as community outreach in varied settings. Encourages students to engage with and learn from their diverse peers, cultivating a collaborative learning environment where different experiences and perspectives can contribute to a deeper, more comprehensive understanding of nutrition and health. Trains health professionals on how to provide personalized nutritional advice that respects each individual’s unique needs and experiences related to their gender identity and biological sex. Provides tools for developing personalized dietary plans that cater to the individual’s specific needs, while also taking into account their cultural preferences, lifestyle, and overall well-being.

**Table 2:** DEIB in Recipes and Cooking.

Cultural Representation and Respect	Ensures that cultural recipes are represented accurately and with respect. Provides context or background information on the cultural significance of the dish. Includes recipes from a diverse range of cultures. Provides historical context or background information for traditional or cultural dishes. Credits the sources or cultural roots of recipes, especially if they are borrowed or inspired by a particular culture or tradition. Educates learners on the origins and significance of ingredients and cooking methods. Ensures authenticity and cultural sensitivity through community input. Encourages the recognition and celebration of food as a vital part of a person’s heritage and ethnicity. Encourages consideration of not just the nutritional aspects of food, but also the social, emotional, and spiritual dimensions of eating, reflecting a holistic understanding of dietary practices within various cultural contexts. Collaborates with individuals from diverse cultural backgrounds in creating and reviewing recipes. Promotes mindfulness when discussing or engaging with foods and customs from different cultures, emphasizing respectful curiosity and open-mindedness to avoid inadvertent misrepresentation or stereotyping. Encourages users to share their own recipes and cultural dishes.
Accessibility	Considers the accessibility and affordability of ingredients in recipes. Offers alternative ingredients that are more readily available or cost-effective. Explains specific cooking techniques that might not be familiar to everyone in easy-to-understand language. Ensures that recipes are articulated with specificity and clarity, making them accessible and easy to understand for diverse learners. Suggests alternative tools or methods if specialized equipment is required. Considers the need for adaptive tools and techniques to assist individuals with disabilities, making cooking more accessible and comfortable. Provides recipes with adaptable portion sizes to cater to individual needs, promoting shared meals and reducing food waste. Teaches how to interpret food labels and comprehend front-of-package claims, enhancing accessibility and understanding of packaged food products.
Dietary Inclusion	Includes options for various dietary needs (vegetarian, vegan, halal, kosher, gluten-free, lactose-free, allergy-friendly). Clearly labels recipes based on dietary categories. Ensures that recipes can accommodate ingredient substitutions and alternative cooking methods. Provides nutritional information, where applicable. Offers flexibility in portion sizes to cater to different family sizes and nutritional needs.
Language Sensitivity	Uses inclusive and respectful language in recipe descriptions. Avoids language that could be perceived as judgmental or pejorative. Utilizes specific terms to describe food that reflect its origin, ingredients, and cultural influences.

**Table 3:** DEIB in Curriculum Delivery.

Safe Space for Dialogue	Provides guidelines for respectful, inclusive discussions among health professionals. Has a plan in place for addressing discriminatory or offensive language or behavior.
Goal Setting and Evaluation	Has clear, measurable learning objectives that align with DEIB principles and are relevant to health professionals. Utilizes an evaluation method to assess whether objectives are met and how effectively DEI principles have been incorporated into practice.
Continuous Improvement	Has a plan for periodic review and revision to ensure alignment with DEIB best practices in healthcare. Has a structured process for incorporating feedback from health professionals in real-time.
Instructional Methods and Accessibility	Utilizes a variety of teaching methods catering to different learning styles of health professionals. Includes interactive and experiential learning opportunities, such as case studies and role-plays. Measures are in place to ensure that health professionals with limited digital access or literacy can participate. Online content is compatible with assistive technologies. In-person sessions are accessible to health professionals from different socio-economic backgrounds. Provides materials accessible to health professionals with disabilities.

## Data Availability

No new data were created or analyzed in this study. Data sharing is not applicable to this article.
